# The evolving role of immune checkpoint inhibitors in cervical and endometrial cancer

**DOI:** 10.20517/cdr.2023.120

**Published:** 2024-06-11

**Authors:** Bertha Alejandra Martinez-Cannon, Ilaria Colombo

**Affiliations:** ^1^Hematology-Oncology Department, National Institute of Medical Sciences and Nutrition Salvador Zubiran, Mexico City 14080, Mexico.; ^2^Medical Oncology, Oncology Institute of Southern Switzerland (IOSI), Ente Ospedaliero Cantonale (EOC), Bellinzona 6500 - CH, Switzerland.

**Keywords:** Immunotherapy, cervical cancer, endometrial cancer, immune checkpoint inhibitors

## Abstract

The introduction of immune checkpoint inhibitors (ICIs) has revolutionized the treatment landscape for numerous tumor types, including cervical and endometrial cancers. Multiple ICIs against programmed cell death-1 (PD-1), programmed death-ligand 1 (PD-L1), and cytotoxic T lymphocyte-associated antigen 4 (CTLA-4) have demonstrated encouraging outcomes in controlled clinical studies for advanced cervical and endometrial cancers. For advanced cervical cancer, approved ICIs as second-line treatment include cemiplimab, nivolumab, and pembrolizumab as single agents. In the first-line treatment setting, options include pembrolizumab alone or in combination with bevacizumab, as well as atezolizumab combined with a backbone platinum-based chemotherapy plus bevacizumab. Additionally, for locally advanced cervical cancer, pembrolizumab is recommended alongside concurrent chemoradiotherapy. For endometrial cancer, pembrolizumab monotherapy, pembrolizumab in combination with lenvatinib, and dostarlimab are currently approved as second-line treatment options. Moreover, either dostarlimab or pembrolizumab can be added to first-line platinum-based chemotherapy for mismatch repair deficient malignancies. Although the inclusion of these agents in clinical practice has led to improved overall response rates and survival outcomes, many patients still lack benefits, possibly due to multiple intrinsic and adaptive resistance mechanisms to immunotherapy. This review aims to highlight the rationale for utilizing ICIs and their current role, while also delineating the proposed mechanisms of resistance to ICIs in cervical and endometrial cancer.

## INTRODUCTION

Recently, immunotherapy has drastically transformed the management of many solid and hematologic malignancies^[1-5]^. immune checkpoint inhibitors (ICIs) function by blocking inhibitory signals in immune cells that are mediated by the programmed cell death-1 (PD-1), its ligand [programmed death-ligand 1 (PD-L1)], and the cytotoxic T cell lymphocyte-associated antigen 4 (CTLA-4), which typically hinder antitumor immunity when activated, aiming to reinstate the antitumor activity of immune cells^[6-8]^.

In 2017, pembrolizumab was approved as the first tumor-agnostic, histology-independent treatment for second-line therapy of microsatellite instability-high (MSI-H)/mismatch repair deficient (dMMR) malignancies^[9]^. In this phase II study involving 86 patients with advanced MSI-H/dMMR solid tumors, 21% experienced a complete response (CR), reaching an objective response rate (ORR) of 53%. Notably, the 15 patients with endometrial cancer (EC) included in this trial presented similar outcomes to those of the overall trial population. Then, in 2020, the phase II KEYNOTE-158 study confirmed the approval of pembrolizumab for non-colorectal MSI-H/dMMR tumors^[10]^. Among the 27 tumor types evaluated in this trial, EC had the highest ORR at 57.1%. These results led to subsequent studies further evaluating the role of ICIs in multiple cancer types, including cervical cancer (CC) and EC.

This review aims to highlight the rationale for employing ICIs and their current role, as well as to describe the proposed mechanisms of resistance to ICIs in CC and EC.

## CERVICAL CANCER

### Rationale for ICIs

CC ranks as the fourth most commonly diagnosed cancer globally and is the fourth leading cause of cancer-related mortality among women^[11]^. In resource-constrained settings, the burden of CC is higher, where access to screening and treatment is limited^[11]^. Despite being among the most preventable cancers, CC still causes a substantial number of cancer deaths in women because of the ineffective treatment options available for women with locally advanced and metastatic CC^[12]^. CC is primarily attributable to infection by the human papillomavirus (HPV), with approximately 70% of cases related to the high-risk genotypes 16 and 18^[13]^. The primary carcinogenic mechanism after HPV infection involves the incorporation of essential HPV oncoproteins (E6 and E7) into the human genome. E6 leads to the inhibition of p53, blocking apoptosis, while E7 inhibits the retinoblastoma tumor suppression protein, leading to cell cycle arrest^[14]^. Furthermore, somatic mutations of the host genome and DNA methylation associated with HPV infection leading to a high mutational tumor burden (TMB) have also been described as an essential aspect of CC oncogenesis^[15-18]^. Finally, approximately 88% of locally advanced CC are PD-L1-positive with a cut-off value of ≥ 1% as assessed by immunohistochemistry on tumor cells, and 96% of cases exhibit some degree of PD-L1 staining (> 0% positive staining within the tumor)^[19]^. These mechanisms provide rationale regarding the immunogenicity of CC and suggest the potential role of ICIs in this gynecological malignancy.

### Advanced cervical cancer

#### Second-line setting

**Figure 1:**
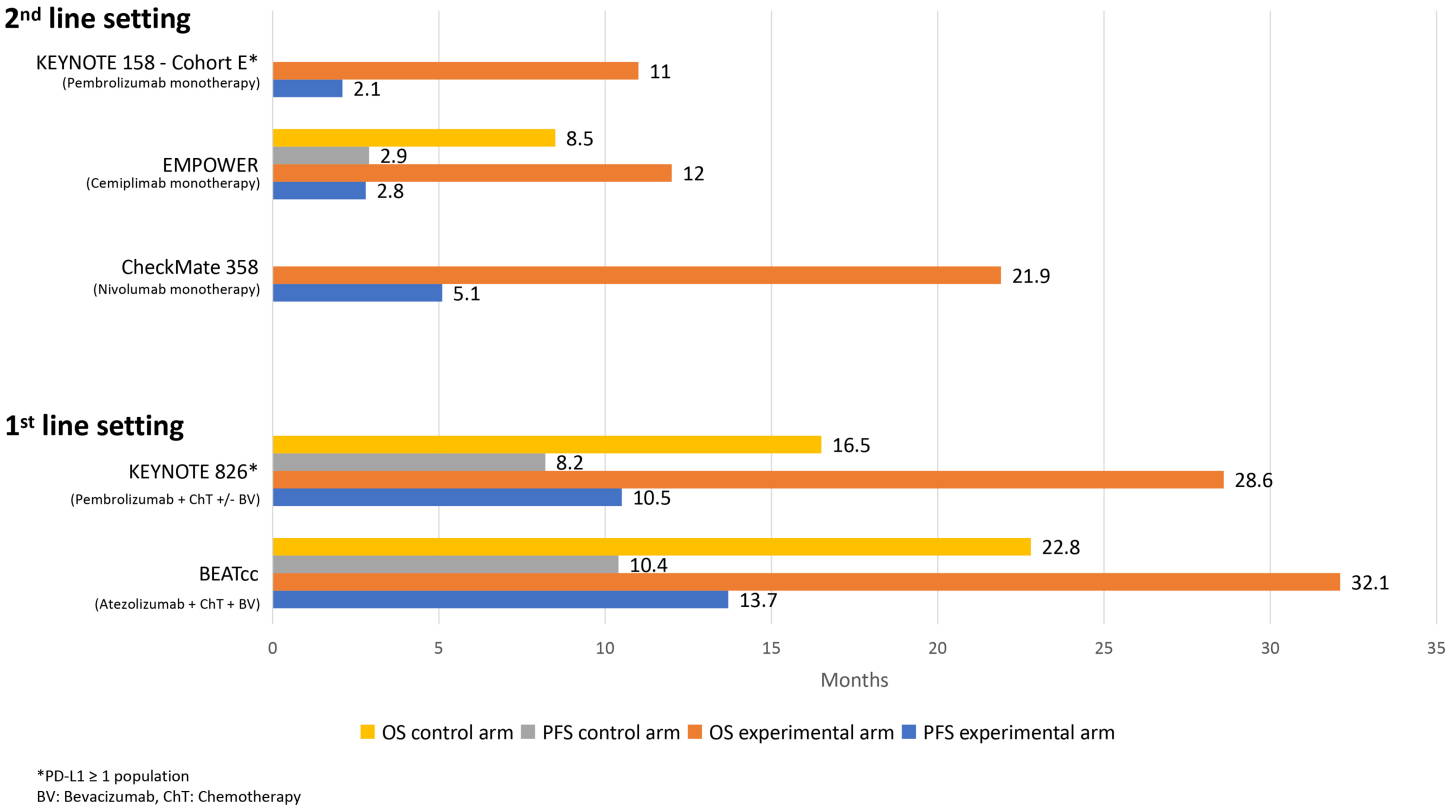
Overall and progression-free survival for ICI alone or plus chemotherapy for the treatment of advanced/recurrent CC. ICI: Immune checkpoint inhibitor; CC: cervical cancer.

Several trials have demonstrated encouraging activity and survival benefits with the employment of ICIs for advanced and/or recurrent CC, leading to the inclusion of these agents in clinical practice guidelines and approval for their use by international regulatory agencies [Figure 1].

The phase II KEYNOTE-158 study assessed the activity of pembrolizumab across multiple cancers, including 98 patients with previously treated advanced CC (Cohort E)^[20]^. After a median follow-up period of 36.9 months, the ORR was 14.3%, and all patients had a PD-L1 combined positive score (CPS) score ≥ 1. The entire study population’s median progression-free survival (PFS) and median overall survival (OS) were 2.1 and 9.3 months, respectively^[21]^.

In the randomized phase III EMPOWER-Cervical 1/GOG-3016/ENGOT-cx9 trial, 608 women with metastatic CC that had progressed following platinum-based chemotherapy were randomized to receive either cemiplimab monotherapy or chemotherapy selected by the investigator^[22]^. The study achieved its primary endpoint, showing a median OS of 12 months for the cemiplimab group compared to 8.5 months for the standard of care arm [hazard ratio (HR) 0.69; 95% confidence interval (CI) 0.56-0.84; *P* < 0.001]. This survival benefit was observed independently of the histological subtype and PD-L1 status. The ORR (16.4% compared to 6.3%; *P* < 0.001) and the median PFS (2.8 months compared to 2.9 months; HR 0.75; 95%CI 0.63-0.89; *P* < 0.001) were also improved with cemiplimab. Moreover, patients with PD-L1 ≥ 1% who received cemiplimab demonstrated longer OS and higher ORR. Overall, adverse events (AEs) grade ≥ 3 were observed in 45% of the patients in the cemiplimab group and 53.4% of those in the control group, with a lower frequency of grade ≥ 3 anemia (12% *vs.* 27%) and grade ≥ 3 neutropenia (1% *vs.* 9%) in patients treated with immunotherapy. These results led to the approval of cemiplimab for the treatment of CC by the European Medicines Agency (EMA).

The phase I/II trial CheckMate 358 explored the efficacy of nivolumab alone and in combination with ipilimumab in treating virus-associated cancers, including patients with recurrent/metastatic CC (≤ 2 prior lines of systemic therapy)^[23]^. Patients were randomized to receive either nivolumab 240 mg every two weeks, nivolumab 3 mg/kg every two weeks and ipilimumab 1 mg/kg every six weeks (N3+I1), or nivolumab 1 mg/kg and ipilimumab 3 mg/kg every three weeks for four cycles, followed by nivolumab 240 mg every two weeks for two years (N1+I3), or until disease progression, unacceptable side effects, or withdrawal of consent^[24]^. ORR was 26% for nivolumab monotherapy, 31% for N3+I1, and 40% for N1+I3, irrespective of PD-L1 status. The DOR was not reached with nivolumab monotherapy, 24.4 months with the N3+I1 regimen, and 34.1 months with the N1+I3 regimen. Median OS was 21.6, 15.2, and 24.7 months, respectively. The frequency of grade ≥ 3 immune-mediated AEs was < 6% for nivolumab monotherapy, < 7% for N3+I1, and < 6% (except for hepatitis 16%) for N1+I3. These findings indicated that chemotherapy-free treatment regimens with nivolumab, either alone or combined with ipilimumab, provided sustained responses with manageable AEs in patients^[25]^.

A phase II trial, conducted in an open-label manner, evaluated the efficacy of a combination therapy consisting of balstilimab (an anti-PD1 agent) and zalifrelimab (an anti-CTLA-4 agent) as second-line treatment for women with advanced CC^[26]^. Among 125 women with measurable disease, ten patients achieved complete responses and 22 partial responses, reaching an ORR of 25.6%, with 64.2% of patients sustaining a response at 12 months. Among women with tumors showing PD-L1 expression, the ORR reached 32.8%, while 9.1% for those lacking PD-L1 expression. The predominant AEs were low-grade (G1-2), including hypothyroidism, diarrhea, fatigue, and nausea. The overall incidence of (G ≥ 3) AEs was 20%. Therefore, this combination proved to be a promising regimen with enduring clinical activity and manageable toxicity in women with advanced CC.

#### First-line setting

The phase III trial KEYNOTE-826, a randomized, placebo-controlled study, investigated the incorporation of pembrolizumab into platinum-based chemotherapy, with or without bevacizumab, as first-line treatment in 617 women with persistent, recurrent, or metastatic CC^[27]^. The study demonstrated that adding pembrolizumab improved PFS and OS, its dual primary endpoints. In the intention-to-treat analysis, the pembrolizumab group showed a median PFS of 10.4 months compared to 8.2 months in the placebo group (HR 0.61; 95%CI 0.50-0.74; *P* < 0.001), and a median OS of 26.4 months compared to 26.8 months in the placebo group (HR 0.63; 95%CI 0.52-0.77; *P* < 0.001). In patients with PD-L1 positive tumors, the pembrolizumab arm demonstrated a median PFS of 10.5 months *vs.* 8.2 months in the placebo arm (HR 0.57; 95%CI 0.47-0.71; *P* < 0.001) and a median OS of 28.6 months compared to 16.5 months in the placebo arm (HR 0.60; 95%CI 0.49-0.74; *P* < 0.001)^[28]^. All protocol-specified subgroups for PFS and OS, including age, race, ECOG, PD-L1 status, bevacizumab use, and stage at diagnosis, favored the pembrolizumab group. Grade ≥ 3 AEs occurred in 82% of the study population treated with pembrolizumab and 75% in the placebo group. Among the most common grade ≥ 3 AEs were anemia, affecting 30% in the pembrolizumab group and 27% in the placebo group, and neutropenia observed in 12% and 10%, respectively. The outcomes of the KEYNOTE-826 trial demonstrated significant improvements in both PFS and OS by incorporating pembrolizumab into platinum-based chemotherapy with or without bevacizumab for patients with advanced CC as first-line treatment, leading to regulatory approval.

Finally, the recently published BEATcc study evaluated adding atezolizumab to platinum-based chemotherapy and bevacizumab among 410 patients with previously untreated metastatic, persistent, or recurrent CC^[29]^. Patients were randomized to receive standard platinum-based chemotherapy and bevacizumab with or without atezolizumab. The study showed a PFS of I 13.7 months in the experimental arm compared to 10.4 months in the standard arm (HR 0.62; 95%CI 0.49-0.78; *P* < 0.0001). Moreover, the median OS was 32.1 months in the atezolizumab group *vs.* 22.8 months in the chemotherapy group (HR 0.68; 95%CI 0.52-0.88; *P* = 0.0046). Although the incidence of grade ≥ 3 AEs was similarly high in both groups, affecting 79% of patients in the atezolizumab group and 75% in the chemotherapy arm, grade ≤ 2 diarrhea, arthralgia, pyrexia, and rash were higher among patients in the atezolizumab group. Thus, incorporating atezolizumab into standard platinum-based chemotherapy with bevacizumab plus for metastatic, persistent, or recurrent CC significantly improved PFS and OS and could be another first-line treatment option.

### Locally advanced cervical cancer

Given the clinically meaningful survival benefit observed with immunotherapy-containing regimens in the advanced setting, incorporating these agents is currently being investigated in the locally advanced setting. The efficacy and safety of adding durvalumab during and after concurrent chemoradiotherapy (CCRT) was evaluated in the phase III randomized CALLA trial among women with locally advanced CC. Initial results indicated that adding durvalumab to CCRT did not result in a statistically significant improvement in PFS (HR 0.84; 95%CI 0.65-1.08; *P* = 0.174) or OS (HR 0.78; 95%CI 0.65-1.10; *P* = 0.156) compared to CCRT alone^[30]^.

The ENGOT-cx11/KEYNOTE-A18 trial investigated the role of adding pembrolizumab concurrently and following CCRT in 1,060 women with high-risk locally advanced CC and assessed different pembrolizumab regimens administered concurrently with and following CCRT^[31]^. The primary outcomes were PFS and OS. After a median follow-up of 17.9 months, the 24-month PFS rate was 68% in the pembrolizumab-CCRT arm compared to 57% in the placebo-CCRT arm (HR 0.70; 95%CI 0.55-0.89; *P* = 0.0020). The 24-month OS rate was 87% in the pembrolizumab-CCRT arm and 81% in the placebo-CCRT arm (HR 0.73; 95%CI 0.49-1.07). Grade ≥ 3 AEs were recorded at 75% in the pembrolizumab-CCRT arm and 69% in the placebo-CCRT arm.

The negative outcome of the CALLA study compared to the positive results of KEYNOTE-A18 may be attributed to several factors, including patient characteristics, PD-L1 status, sample sizes, and follow-up. Firstly, 66% of women in the CALLA study had FIGO 2009 III-IVA stage and 74% node-positive disease, whereas in the KEYNOTE-A18 trial, these proportions were 56% and 84%, respectively. Additionally, disparities in PD-L1 positivity rates may have contributed. A post hoc subgroup analysis in the CALLA trial showed that women expressing higher PD-L1 tumor area positivity had a lower risk of progression with durvalumab treatment. Conversely, KEYNOTE-A18 did not show enrichment in treatment effect based on PD-L1 positivity. Furthermore, differences in sample sizes and follow-up durations could have played a role. CALLA had a smaller sample size (*n* = 770) and longer median follow-up (18.5 months) compared to KEYNOTE-A18 (*n* = 1,060, 17.9 months). While cross-trial comparisons should be approached cautiously, these factors highlight the importance of interpreting results within the context of trial design and participant demographics.

Finally, the ATOMICC trial, a currently ongoing, randomized, open-label, phase II study, is assessing the activity of TSR-042 (anti-PD1) as maintenance therapy for women with high-risk locally advanced CC following CCRT^[32]^.

## ENDOMETRIAL CANCER

### Rationale for ICIs

EC is the most frequent gynecologic malignancy^[11]^. Almost two-thirds of women diagnosed with EC present with stage I disease, with 5-year OS rates reaching approximately 95%^[33]^. In the advanced setting, standard first-line chemotherapy provides limited benefit, with a PFS of only 13 months^[34]^. EC is classified into four molecular subtypes: polymerase ε (POLE) mutant (ultramutated), MSI-H (hypermutated), copy number low, and copy number high^[35]^. POLE-mutated and MSI-H tumors represent approximately 40% of all EC cases among these subtypes. They are considered to have high genomic instability and immunogenic phenotypes, harboring more tumor-specific neoantigens and increased amounts of tumor-infiltrating lymphocytes (TILs), resulting in upregulation of compensatory immune checkpoint mechanisms and the overexpression of PD-1 and PD-L1^[36,37]^. These findings led to the investigation of ICIs as a possible treatment for EC.

### Recurrent, advanced, and metastatic endometrial cancer

#### Second-line setting

**Figure 2:**
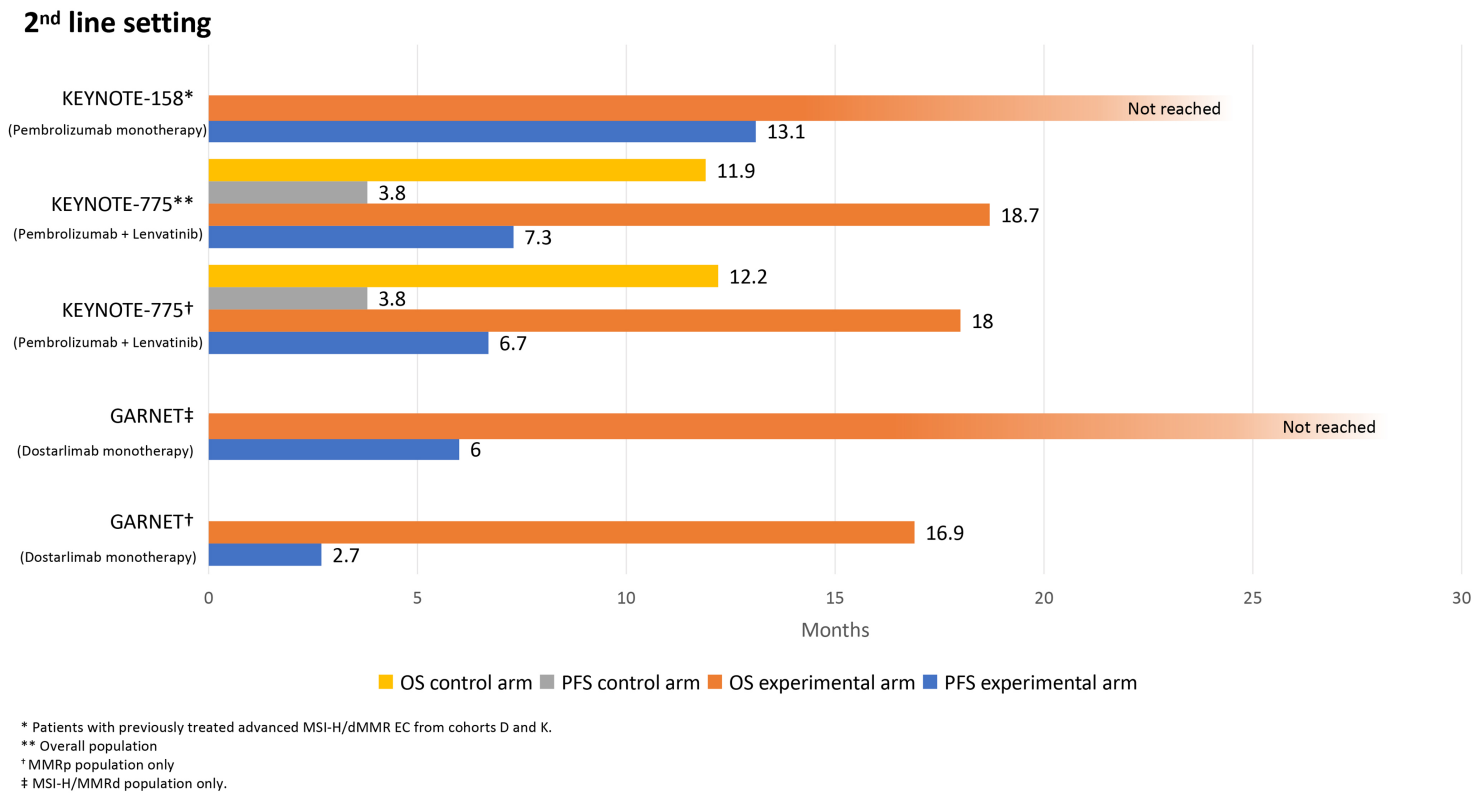
Overall and progression-free survival for ICI alone or plus lenvatinib in the second-line treatment of EC. ICI: Immune checkpoint inhibitor; EC: endometrial cancer.

Multiple trials have demonstrated encouraging activity and survival benefits with ICIs in this setting, resulting in the approval of these agents for routine clinical practice [Figure 2].

ICIs as single-agent

 Pembrolizumab was examined across various advanced cancers in the phase II KEYNOTE-158 basket study. Patients diagnosed with EC regardless of MSI status (Cohort D) and patients with any MSI-H/dMMR advanced solid tumor (excluding colorectal cancer), including EC (Cohort K), were enrolled^[38]^. In the 90 patients with MSI-H/dMMR EC subgroup, the ORR was 48% with a median PFS of 13.1 months. Additionally, the median DOR and OS were not reached. Although 76% of the AEs were of any grade, only 12% were grades 3-4. Thus, pembrolizumab demonstrated high clinical activity with promising survival benefits and manageable toxicity among previously treated women with advanced MSI-H/dMMR EC. Following these findings, the Food and Drug Administration (FDA) granted approval for pembrolizumab monotherapy for individuals with advanced MSI-H/dMMR EC who experienced disease progression following previous systemic therapy.

The open-label phase I GARNET study investigated dostarlimab (500 mg every three weeks for four cycles, followed by 1,000 mg every six weeks) in individuals with advanced solid tumors. This trial included two cohorts of patients diagnosed with EC: those with MSI-H/dMMR tumors (cohort A1, *n* = 153) and those with microsatellite stable (MSS)/mismatch repair proficient (pMMR) tumors (cohort A2, *n* = 161)^[39]^. In cohort A1, the ORR was 45.5%, with 23 complete responses and 42 partial responses, whereas in cohort A2, the ORR was notably lower at 15.4%, with only four complete responses. The median DOR was not reached in cohort A1 (38.9 months to not reached) and was 19.4 months (8.2 months to not reached) in cohort A2. The median PFS was 6.0 months (4.1 to 18.0 months) for cohort A1 and 2.7 months (2.6 to 2.8 months) for cohort A2. Regarding OS, the median was not reached (ranging from 27.1 months to not reached) in cohort A1, while cohort A2 was 16.9 months (ranging from 13 to 21.8 months). In both cohorts, 80.9% of patients presented grade 1-2 AEs, the most frequent being fatigue, diarrhea, and nausea, and 19.1% of patients experienced a grade 3 AE. Hence, dostarlimab demonstrated sustained response rates in women with both MSI-H/dMMR and MSS/pMMR EC and a manageable safety profile. As a result of these findings, dostarlimab was authorized by EMA and FDA for the treatment of patients with recurrent or advanced EC expressing MSI-H/dMMR after progression to platinum-based chemotherapy.

ICIs plus multikinase inhibitors

 While MSI-H/dMMR EC accounts for only 16% of recurrent disease cases^[40]^, and responses and survival benefits with immunotherapy alone have been limited in patients with MSS/pMMR disease^[41,42]^, recent studies have uncovered possible strategies to overcome this challenge. The vascular endothelial growth factor (VEGF) fosters tumor proliferation and aids in metastasis across various tumor types, by stimulating angiogenesis^[43]^. Blocking VEGF may effectively overcome ICI resistance through vascular normalization, and recruitment and proliferation of immune-suppressing cells, including myeloid and regulatory T cells^[44]^. The KEYNOTE-146/Study 111, a phase Ib/II clinical trial, assessed the combination of pembrolizumab and lenvatinib, showing promising results in patients with EC^[45]^.

In the phase III KEYNOTE-775 study, individuals with advanced EC who had undergone ≥ 1 previous line of platinum-based chemotherapy were randomly assigned to either lenvatinib (20 mg daily) alongside pembrolizumab (200 mg administered every three weeks) or the investigator’s choice of chemotherapy (doxorubicin or paclitaxel)^[46]^. The two primary outcomes of PFS and OS were assessed in all comers and patients with pMMR tumors. Out of the 827 women enrolled, 697 were diagnosed with pMMR tumors, while 130 had dMMR tumors. Among those with pMMR tumors, median PFS was 6.7 and 3.8 months with pembrolizumab-lenvatinib and chemotherapy, respectively (HR 0.60; 95%CI 0.50-0.72). Similarly, the median OS was 18.0 months with lenvatinib and pembrolizumab *vs.* 12.2 months with physician’s choice chemotherapy (HR 0.70; 95%CI 0.58-0.83). In the entire study population, both median PFS (7.3 *vs.* 3.8 months, HR 0.56; 95%CI 0.48-0.66) and OS (18.7 *vs.* 11.9 months, HR 0.65; 95%CI 0.55-0.77) were significantly longer in the experimental arm. Grade ≥ 3 AEs occurred more frequently in the pembrolizumab and lenvatinib arm (90.1%) compared to the chemotherapy arm (73.7%)^[47]^. Hence, the combined use of lenvatinib and pembrolizumab enhanced both PFS and OS in all women with advanced EC as a second-line treatment, encompassing those with pMMR disease. These outcomes prompted the EMA approval of pembrolizumab in conjunction with lenvatinib for treating advanced or recurrent EC in patients who have received ≥ 1 line of platinum-based chemotherapy. Furthermore, the FDA approved this combination in the second-line setting for patients with MSI-H/dMMR EC.

ICIs plus poly (ADP-ribose) polymerase inhibitors

Combining ICIs with poly (ADP-ribose) polymerase (PARP) inhibitors (PARPi) may be another treatment option, as these agents have been demonstrated to augment PD-L1 expression and neoantigen burden in preclinical studies^[48]^. The investigator-initiated, multicenter, phase II DOMEC trial examined the efficacy and safety of combining durvalumab with olaparib in women with metastatic or recurrent EC who had received ≥ 1 previous line of platinum-based chemotherapy or were not able or unwilling to undergo chemotherapy^[49]^. PFS at six months was 34% (17/50 patients), and ORR was 16% with only one complete response. The median PFS and OS were 3.4 and 8.0 months, respectively.

Similarly, another investigator-initiated, open-label, single-arm, phase II study evaluated the potential efficacy and safety of combining avelumab with talazoparib in recurrent pMMR EC^[50]^. The co-primary endpoints consisted of ORR and 6-month PFS. Among 35 patients analyzed, the ORR was 11.4%, with four patients achieving partial responses, and the 6-month PFS rate was 22.9%. The most frequently reported grade ≥ 3 AEs included anemia (46%), thrombocytopenia (29%), and neutropenia (11%). Patients with homologous recombination deficiency (HRD)-positive tumors demonstrated higher rates of clinical benefit and longer PFS than those with HRD-negative tumors. PD-L1 status, TMB, and TILs were not associated with clinical benefits from avelumab plus talazoparib.

#### First-line setting

ICIs plus chemotherapy

**Figure 3:**
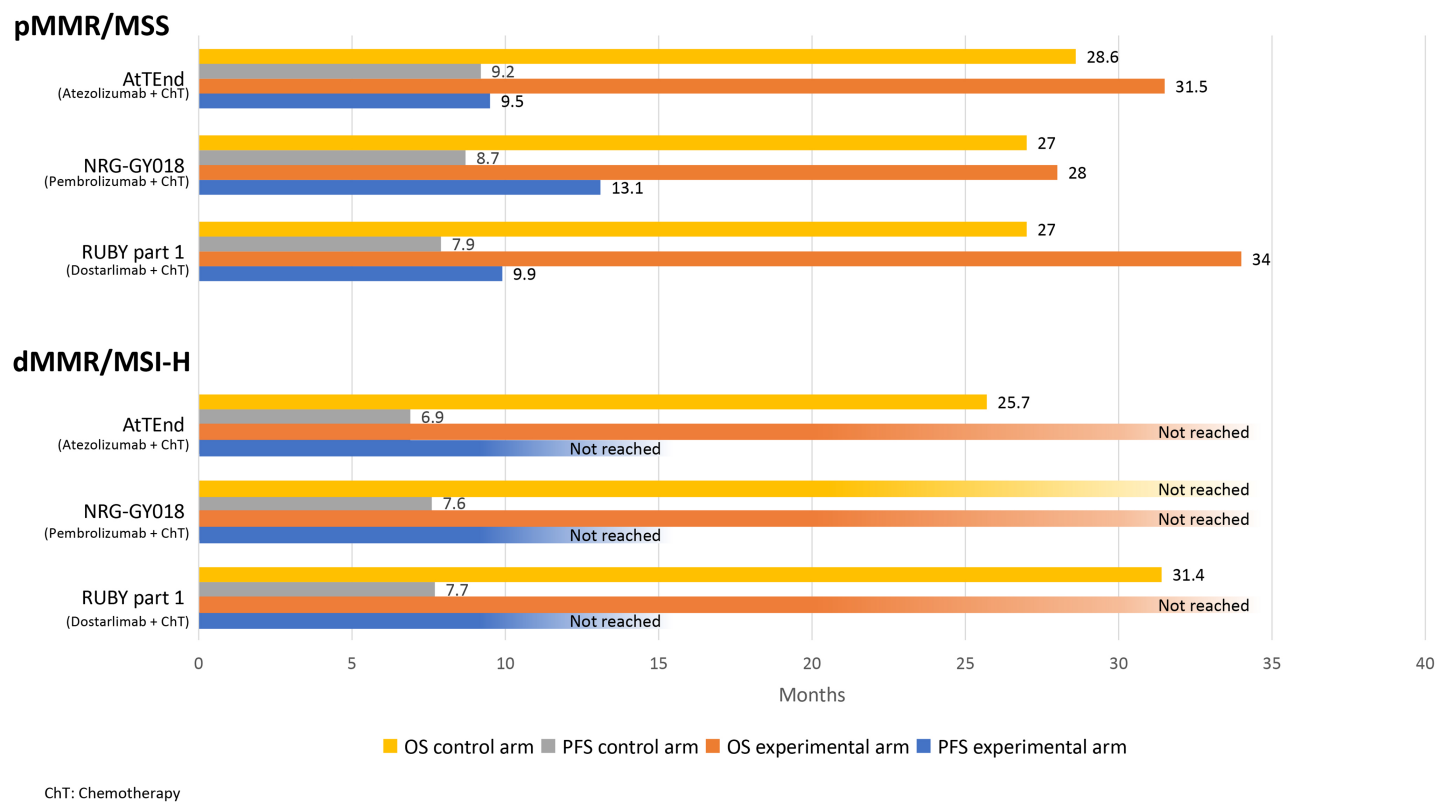
Overall and progression-free survival with ICI plus chemotherapy for the first-line treatment of advanced/recurrent EC. ICI: Immune checkpoint inhibitor; EC: endometrial cancer.

The promising outcomes from studies examining the efficacy of ICIs in the second-line setting prompted numerous trials investigating various ICIs in the first-line [Figure 3]. In part 1 of the phase III RUBY trial, 494 women with stage III-IV or recurrent EC were randomly assigned to receive either dostarlimab or placebo, in addition to standard carboplatin and paclitaxel, followed by maintenance therapy with either dostarlimab or placebo for up to three years^[51]^. Among the MSI-H/dMMR population, a statistically significant and clinically meaningful improvement in PFS was reported (not reached in the dostarlimab group *vs.* 7.7 months in the placebo group; HR 0.28; 95%CI 0.16-0.50). In the overall population, the median PFS was 11.8 months for those receiving dostarlimab and 7.9 months for those on placebo (HR 0.64; 95%CI 0.51-0.80; *P* < 0.001)^[52]^. In the MSS/pMMR population, the estimated 24-month PFS was higher with dostarlimab (28.4%) compared to placebo (18.8%), though the advantage appeared smaller (HR 0.76; 95%CI 0.59-0.98). Additionally, after a median follow-up of 37.2 months, the median OS in the overall population was 44.6 months with dostarlimab *vs.* 28.2 months with placebo (HR 0.69; 95%CI 0.54-0.89; *P* = 0.002). In the dMMR/MSI-H group, the median OS was not reached in the dostarlimab arm, while it was 31.4 months in the placebo arm (HR 0.32; 95%CI 0.17-0.63; *P* = 0.002). In the pMMR/MSS population, the median OS was 34 months for those treated with dostarlimab compared to 27 months for those receiving placebo (HR 0.79; 95%CI 0.60-1.04; *P* = 0.049)^[53]^. The most frequent AEs were comparable between the dostarlimab and placebo groups.

The NRG-GY018 trial enrolled 816 women with measurable stage III or IVA disease, or stage IVB or recurrent EC, to receive pembrolizumab or placebo alongside paclitaxel and carboplatin every three weeks for six cycles, followed by up to 14 additional cycles every six weeks^[54]^. Similarly to the RUBY Part 1 study, an unprecedented benefit in the dMMR group treated with the addition of the ICI was reported (12-month PFS of 74% *vs.* 38%; HR 0.30; 95%CI 0.19-0.48; *P* < 0.001). For the pMMR group, the median PFS was 13.1 months for the pembrolizumab group *vs.* 8.7 months for the placebo group (HR 0.54; 95%CI 0.41-0.71; *P* < 0.001). Adding pembrolizumab to chemotherapy did not result in increased toxicity, and the occurrence of immune-mediated AEs was consistent with those observed in previous studies.

In the AtTEnd phase III study, 551 women with either advanced/metastatic or recurring EC were randomly assigned (2:1 ratio) to carboplatin and paclitaxel with the anti-PD-L1 atezolizumab or placebo, followed by maintenance until disease progression^[55]^. In the dMMR subgroup, the addition of atezolizumab resulted in improved PFS (HR 0.36; 95%CI 0.23-0.57; *P* = 0.0005), with the median PFS not being reached in the atezolizumab arm *vs.* 6.9 months in the placebo group. In the overall population, the median PFS was 10.1 months for the atezolizumab group compared to 8.9 months for the placebo group (HR 0.74; 95%CI 0.61-0.91; *P* = 0.0219). Grade ≥ 3 AEs were observed in 66.9% of patients in the atezolizumab arm compared to 63.8% in the placebo arm.

The outcomes from the aforementioned trials have prompted shifts in the management of advanced EC in the first-line setting. Nonetheless, the ongoing KEYNOTE-C93 (NCT05173987) and DOMENICA (NCT05201547) trials are investigating monotherapy ICI approaches in patients with dMMR/MSI-H EC.

ICIs plus multikinase inhibitors

Given the proven efficacy of pembrolizumab and lenvatinib in the second-line setting, this combination was also investigated in the first-line setting in the phase III ENGOT-en9/LEAP-001 trial^[56]^. Participants were randomized to receive lenvatinib 20 mg daily along with pembrolizumab 200 mg every three weeks or to undergo treatment with paclitaxel at a dose of 175 mg/m^2^ plus carboplatin at an AUC 6 administered every three weeks. The primary endpoints were PFS and OS evaluated in both the pMMR and intention-to-treat populations. After a median follow-up duration of 38.4 months, in the pMMR subgroup, the comparison between lenvatinib plus pembrolizumab and chemotherapy did not show statistically significant non-inferiority for OS (HR 1.02; 95%CI 0.83-1.26; non-inferiority *P* = 0.2459875). In patients with advanced or recurrent EC, the first-line treatment with lenvatinib and pembrolizumab did not meet the predetermined statistical criteria for either OS or PFS compared to chemotherapy. However, the safety profile continued to be manageable and in line with that previously observed with this combination.

ICIs plus PARP inhibitors

The DUO-E was a 3-arm phase III trial in patients with advanced or recurrent EC, investigating the combination of carboplatin and paclitaxel alongside durvalumab, followed by maintenance treatment with durvalumab with or without olaparib in the first-line setting^[57]^. In the intention-to-treat analysis, both the durvalumab and the durvalumab plus olaparib arms exhibited statistically significant improvements in PFS compared to the control arm. Specifically, the durvalumab arm showed a PFS of 10.2 months compared to 9.6 months in the control group (HR 0.71; 95%CI 0.57-0.89; *P* = 0.003), while the durvalumab plus olaparib arm demonstrated a PFS of 15.1 months compared to 9.6 months in the control arm (HR 0.55; 95%CI 0.43-0.69; *P* < 0.0001). In prespecified, exploratory subgroup analyses, a PFS benefit was observed in the dMMR population, with HRs of 0.42 (95%CI 0.22-0.80) in the durvalumab arm and 0.41 (95%CI 0.21-0.75) in the durvalumab plus olaparib arms compared to the control arm. Similarly, in the pMMR population, a PFS benefit was also demonstrated, with HRs of 0.77 (95%CI 0.60-0.97) in the durvalumab arm and 0.57 (95%CI 0.44-0.73) in the durvalumab plus olaparib arm compared to the control arm. The interim OS results, with approximately 28% maturity, indicated a trend toward enhanced OS with durvalumab (HR 0.77; 95%CI 0.56-1.07; *P* = 0.120) and durvalumab plus olaparib (HR 0.59; 95%CI 0.42-0.83; *P* = 0.003) compared to the control group.

Finally, in RUBY part 2, the efficacy and safety of dostarlimab in combination with carboplatin-paclitaxel, followed by dostarlimab and niraparib, was assessed in 291 women diagnosed with recurrent or advanced EC^[58]^. With a median follow-up of 19 months, the median PFS was 14.5 months in the dostarlimab-niraparib group compared to 8.3 months in the chemotherapy-alone group (HR 0.60; 95%CI 0.43-0.82; *P* = 0.0007). In the MSS/pMMR subgroup, the median PFS was 14.3 months in the dostarlimab-niraparib arm and 8.3 months in the chemotherapy-alone arm (HR 0.63; 95%CI 0.44-0.91; *P* = 0.006). In an exploratory analysis of the MSI-H/dMMR population, the median PFS was not reached in the dostarlimab-niraparib arm, while it was 7.9 months in the chemotherapy-alone arm (HR 0.48; 95%CI 0.24-0.96; *P* = 0.0174). Grade ≥ 3 AEs were documented in 84% of patients in the dostarlimab group and 49% in the placebo group. The most common events included nausea (59.7% *vs.* 50%), fatigue (52.4% *vs.* 42.7%), and anemia (49.7% *vs.* 47.9%).

#### Treatment after progression to ICIs

Data regarding treatment after progression to ICIs among patients with advanced EC are scarce. A phase II clinical trial assessed the activity of combining nivolumab and cabozantinib, a multitargeted tyrosine kinase inhibitor known for its potent activity against VEGF receptor 2, in 20 individuals with EC who experienced disease progression following immunotherapy^[59]^. Combining cabozantinib and nivolumab as ICI rechallenge resulted in an ORR of 25%, with seven patients achieving stable disease, leading to a median DOR of 5.5 months. However, the best treatment approach after the failure of ICIs in EC has yet to be defined, and many trials assessing new agents and strategies are ongoing.

### Localized endometrial cancer

Following the important positive results of using ICI with chemotherapy for the treatment of advanced/recurrent EC, its role has been investigated in the adjuvant setting. Particularly, pembrolizumab is under investigation in combination with adjuvant chemotherapy, with or without radiotherapy, in women with newly diagnosed, high-risk EC (ENGOT-en11/GOG-3053/KEYNOTE-B21 trial). This study is designed with dual primary endpoints, encompassing disease-free survival and OS. Enrollment for this trial started in December 2020 and is presently ongoing^[60]^.

Furthermore, the Refining Adjuvant treatment IN endometrial cancer Based On molecular features (RAINBO) program is investigating four adjuvant treatment strategies tailored to the four molecular subtypes according to the TCGA classification. This program aims to increase cure rates by incorporating novel targeted therapies or safely de-escalating treatment^[61]^. The RED phase III trial is comparing adjuvant CCRT followed by two years of olaparib *vs.* adjuvant CCRT alone in women with p53 abnormal stage I-III EC. In the GREEN phase III trial, adjuvant radiotherapy with concurrent and adjuvant durvalumab for one year is compared to radiotherapy alone in women with dMMR stage II (with lymphovascular space invasion) or stage III EC. The ORANGE segment is a phase III trial aimed at treatment de-escalation for patients lacking a specific molecular profile, focusing on estrogen receptor-positive stage II (with LVSI) or stage III EC and comparing radiotherapy followed by progestin for two years to adjuvant chemoradiation. Lastly, the BLUE part is a phase II trial investigating treatment de-escalation in adjuvant therapy for women with POLE-mutated stage I-III EC, assessing the omission of adjuvant treatment (no adjuvant treatment or radiotherapy alone for higher-risk disease).

## MECHANISMS OF RESISTANCE

Although ORR and survival outcomes have improved with the inclusion of these agents into clinical practice, a substantial percentage of patients with CC and EC still do not experience benefits, possibly due to multiple intrinsic and adaptive resistance mechanisms to immunotherapy^[62]^.

Intrinsic resistance mechanisms to ICIs in CC may include lack of response in pMMR tumors^[63]^, T cell exhaustion due to chronic viral infections^[64]^, and immunosuppressive tumor microenvironment^[65]^. Conversely, patients who initially respond to ICIs may later encounter adaptative resistance, leading to disease progression or recurrence. Adaptive mechanisms of resistance in CC include the upregulation of alternative immune checkpoint signaling pathways^[66]^. Potential targets for maximizing the effect of anti-PD-1 antibodies involve blocking CTLA-4 and T-cell immunoreceptors with immunoglobulin and ITIM domain (TIGIT) co-inhibitory signals, thus creating a synergistic effect^[67,68]^.

The approach of dual checkpoint blockade targeting both PD-1 and CTLA-4 has proven to enhance clinical outcomes compared to sole anti-PD-1 monotherapy across various types of solid tumors^[69]^. Inhibiting PD-1 restores the responsiveness of tumor-reactive T cells^6^ while blocking the CTLA-4 pathway activates effector T cells and diminishes their down-regulating function^[70]^. Therefore, targeting the dual blockade of these separate yet mutually reinforcing mechanisms could effectively counter resistance observed to ICI monotherapy, both in CC and other solid tumors.

Conversely, TIGIT, an immune checkpoint receptor found on both natural killer (NK) cells and T cells, reduces the activity of these immune cells by interacting with its ligand poliovirus receptor (PVR), located on antigen-presenting or tumor cells^[62]^. Furthermore, TIGIT expression in CD8+ T lymphocytes in patients with CC is elevated compared to patients without CC and induces the exhaustion of CD8+ T lymphocytes through NF-κB inhibition and extracellular signal-regulated kinase (ERK) activation, leading to the downregulation of cytokine production. Both *in vivo* and *in vitro* investigations have shown that blocking TIGIT reinstates CD8+ T cells’ ability to produce cytokines and that combining TIGIT and PD-1 inhibitors shows even greater activity than blocking TIGIT alone^[71]^. Currently, ongoing studies are evaluating novel treatment strategies for CC, including combinations of ICIs and adoptive cell therapy to overcome such mechanisms of resistance [Table 1].

**Table 1:** Ongoing trials in advanced cervical cancer

**Trial**	**Phase**	**Novel treatment**	**Setting**	**Primary endpoint**	**Estimated enrollment (*n*)**
**Monotherapy**					
FERMATA (NCT03912415)	III	BCD-100	1st line	OS	316
**Dual checkpoint blockade**					
NCT05033132	II	Balstilimab +/- zalifrelimab	2nd line	PFS	177
RaPiDS (GOG-3028) (NCT03894215)	II	Balstilimab +/- zalifrelimab	2nd line	ORR	200
NCT04380805	II	Cadonilimab (PD-1/CTLA-4 bispecific antibody)	2nd line	ORR	30^*^
NCT04982237	III	Cadonilimab	1st line	PFS and OS	440
**Combination with anti-TIGIT inhibitors**					
KEYVIBE-001 (NCT02964013)	I	Vibostolimab +/- pembrolizumab	2nd line	RP2D	392
SKYSCRAPER-04 (NCT04300647)	II	Atezolizumab +/- tiragolumab	2nd line	ORR	172^*^
**Adoptive cell therapy**					
NCT03108495	II	LN-145	1st and 2nd line	ORR and safety	189

^*^Actual enrollment. OS: Overall survival; PFS: progression-free survival; ORR: objective response rate; PD-1: programmed cell death-1; CTLA-4: cytotoxic T lymphocyte-associated antigen 4; TIGIT: T-cell immunoreceptors with immunoglobulin and ITIM domain.

For EC, some proposed mechanisms for resistance to immunotherapy include the major histocompatibility complex (MHC), heterogenous expression of PD-1, and Janus kinase 1 (JAK1)/signal transducer and activator of transcription 1 (STAT1) mutations^[72]^.

ICIs enhance adaptive antitumor immune responses and require cytotoxic T cells’ presence and activation through various mechanisms, including MHC class I^[73]^. MHC class I loss prevents antigen recognition by neoantigen-specific CD8+ T cells, which may lead to resistance to PD-1 inhibitors regardless of PD-1 expression^[74]^. In a study by Friedman *et al.*, 46% of dMMR and 25% of PD-L1-positive endometrial tumors lost MHC class I expression^[74]^. Thus, further research investigating the role of MHC class I loss on resistance to immunotherapy is needed.

Regarding MHC class II, lymphocyte activation gene-3 (LAG-3), an immune inhibitory receptor found mainly on activated T and NK cells, has been recognized as its primary ligand, hindering the activation of CD4+ helper T cells^[72]^. Studies have demonstrated that fibrinogen-like protein 1 (FGL-1), a critical ligand for LAG-3, can trigger T-cell suppression, facilitating tumor immune evasion^[75]^. Furthermore, *in vitro* experiments have shown that LAG-3 diminishes the activity of CD8+ T cells in the vicinity of tumors and decreases cytokine production through its interaction with galectin-3 (GAL-3).^[76]^ Research has shown that LAG-3 plays a role in evading the immune system’s responses across various solid tumors, including EC^[77]^. In a retrospective study involving 421 EC patients conducted by Zhang *et al.*, LAG-3 expression in immune cells was more prevalent in patients exhibiting high-risk characteristics, including high-grade tumors, those classified in the ESMO-ESTRO-ESGO high-risk group, advanced or metastatic EC, and cases with lymphovascular space invasion. Additionally, higher LAG-3 expression was noted in patients with the POLE-mutated and dMMR molecular subtypes^[77]^. Thus, LAG-3 could potentially be a candidate target for immunotherapy in POLE-mutated and dMMR EC alone or in combination with PD-1/PD-L1 blockade to enhance the immunotherapeutic effect. Nevertheless, additional investigations are required to comprehend the specific mechanisms regulating LAG-3 and its biological significance in EC.

Heterogenous expression of PD-1 within the tumor microenvironment could also sustain resistance against PD-1/PD-L1 antibodies and could potentially be overcome by targeting alternative immune molecules such as T-cell immunoglobulin and mucin domain-containing protein 3 (TIM-3), either in conjunction with or as an alternative to PD-1 blockade^[78]^. TIM-3 is an immune checkpoint present in various immune cell types and holds significant importance in regulating immune responses and tolerance through modulation^[79]^. TIM-3 expressed on Th1, Th17, monocytes, dendritic cells, and macrophages enhances regulatory T cell numbers and contributes to depletion of CD8(+) tumor-infiltrating T cells^[80]^. In a study by Moore *et al.*, immunohistochemistry was performed on 75 endometrial tumors comprising 25 cases with mutL homolog 1 (MLH1) promoter hypermethylation, 25 non-hypermethylated dMMR cases, and 25 pMMR cases to assess the expression of TIM-3 in the tumor and the microenvironment^[81]^. Most cases (77%) displayed TIM-3 tumoral expression of at least 1%. However, dMMR tumors exhibited a higher prevalence of moderate to robust immune cell expression than pMMR cases (66% *vs.* 12%, *P* = 0.00002). These findings propose a possible use for TIM-3 antibodies in a subgroup of patients with EC, including those with pMMR tumors not currently considered for ICI monotherapy.

JAK1 becomes activated by cytokines such as interferon-gamma (IFNγ), influencing various cellular functions, including immune response and cell growth, through the JAK/STAT pathway^[82]^. It has been demonstrated that JAK1 mutant gynecological cancer cell lines lacked the ability to phosphorylate STAT1 tyrosine in response to IFNγ, preventing the stimulation of antigen-processing machinery components, including low molecular weight peptide-2 (LMP2) and transporter associated with antigen processing-1 (TAP1)^[83]^. This impairment in antigen presentation and processing, due to reduced LMP and TAP protein expression, correlates with diminished human leukocyte antigen (HLA) class I upregulation and resistance to cytotoxic T cell-mediated lysis^[84]^. Thus, JAK1/STAT1 mutations lead to declined effector immune cell activation and hindered antigen-presenting mechanisms^[85,86]^. Micro-satellite frameshift insertions and deletions are the critical forms of JAK1/STAT1 mutations present in EC^[87]^ and can be found in approximately 47% of dMMR tumors^[85]^. Hence, assessing these and other potential biomarkers could be pertinent in identifying EC patients who might not benefit from ICIs.

Moreover, the processes related to microsatellite instability could influence responses to ICIs. Microsatellite instability can result from mutations in the *MMR* genes (germline and somatic mutations) and methylation of the *MLH1* gene promoter^[88]^. In a phase II trial assessing pembrolizumab in patients with MSI-H recurrent EC, it was observed that the TMB was significantly higher in Lynch/Lynch-like tumors [median, 2,939 mutations/megabase (Mut/Mb)] compared to sporadic tumors (median, 604 Mut/Mb; *P* = 0.0076). The ORR was 100% in patients with Lynch/Lynch-like disease, contrasting with 44% in sporadic tumors. Additionally, patients with Lynch/Lynch-like EC exhibited improved 3-year PFS and OS rates compared to those with sporadic EC^[89]^. Contrary to these results, a post hoc analysis from patients in cohort A1 of the GARNET trial found no differences in ORR according to Lynch/Lynch-like vs sporadic EC subgroup analysis^[39,90]^. Similarly, an exploratory analysis of the NRG-GY018 study did not prove a different benefit from incorporating pembrolizumab into chemotherapy according to the mechanism of mismatch repair loss^[91]^.

## CONCLUSIONS

ICIs have drastically shifted the treatment and outcomes of women with advanced CC and EC by providing improved response rates and long-lasting benefits in PFS and OS. Several trials have resulted in the approval of multiple treatment choices, including ICI monotherapy and/or combined with targeted agents in the first- and second-line settings of CC and EC. These encouraging results have prompted ongoing trials evaluating these agents in earlier lines and the (neo)adjuvant setting. However, intrinsic and acquired resistance mechanisms to ICIs and how to overcome such resistance still pose a significant unmet need for treating patients with these malignancies. Further research addressing these questions remains a crucial area of active investigation. Finally, identifying predictive biomarkers of response and/or resistance may lead to a better selection of patients with CC and EC who may benefit from these treatments.

## DECLARATIONS

### Copyright

© The Author(s) 2024.
